# Neuroimaging in advanced Parkinson’s disease: insights into pathophysiology, biomarkers, and personalized therapies

**DOI:** 10.1007/s00702-025-02942-y

**Published:** 2025-05-12

**Authors:** Nils Schröter, Sergiu Groppa, Michel Rijntjes, Gabriel Gonzalez-Escamilla, Horst Urbach, Wolfgang H. Jost, Alexander Rau

**Affiliations:** 1 Department of Neurology, Saarland University Medical Center, Building 90, Kirrberger Straße, 66421 Homburg, Germany; 2 Department of Neurology and Clinical Neuroscience, Medical Center, Faculty of Medicine, University of Freiburg, University of Freiburg, Freiburg, Germany; 3 Department of Neuroradiology, Medical Center, Faculty of Medicine, University of Freiburg, University of Freiburg, Freiburg, Germany; 4 Parkinson-Klinik Ortenau, Wolfach, Germany

**Keywords:** Advanced Parkinson’s disease, MRI, PET, Imaging

## Abstract

Advanced Parkinson’s disease (APD) represents a late stage of Parkinson’s disease and is characterized by complex motor and non-motor symptoms that are less responsive to oral dopaminergic therapies. While APD has a relevant impact on patients’ quality of life and requires intensified treatment, consistent diagnostic criteria have only recently been proposed. The precise pathophysiology underlying the symptoms of APD remains poorly understood, making early prognostication and intervention difficult. Neuroimaging has emerged as a promising tool for elucidating the mechanisms driving APD, identifying biomarkers for disease staging, and predicting therapeutic response. Techniques such as molecular imaging and magnetic resonance imaging provide insight into molecular and structural changes associated with the progression of PD, including protein aggregation, neuroinflammation, and regional neurodegeneration. While positron emission tomography imaging of alpha-synuclein and other pathologies offers avenues for staging and differential diagnosis, advanced magnetic resonance imaging approaches have the potential for capturing subtle microstructural changes i.e. through neuromelanin-sensitive or diffusion-weighted imaging. However, the majority of imaging studies has focused on early Parkinson’s disease, leaving their applicability to APD uncertain. Future research should prioritize the validation of neuroimaging findings in well-defined APD cohorts and extend their use to predict clinical milestones such as motor fluctuations, dyskinesia, and cognitive decline. These efforts are essential to advance personalized therapeutic strategies and bridge the gap between research and clinical management of APD.

## Introduction

Advanced Parkinson’s disease (APD) constitutes the late stage in the course of PD and is characterized by complex motor and non-motor symptoms that are less responsive to conventional dopaminergic therapies (Coelho and Ferreira 2012; Jost 2024). This stage usually manifests after long-lasting disease progression, frequently with the presence of motor fluctuations, instability of gait, and levodopa-induced dyskinesia. Moreover, non-motor symptoms such as cognitive impairment, autonomic dysfunction, and neuropsychiatric disturbances, including visual hallucinations, are present and associated with a reduced quality of life. While APD is burdensome for patients and caregivers, unified criteria for diagnosis of APD were only recently proposed by Luquin et al. as the results of a Delphi process for the definition of APD (Luquin et al. 2017). Here, they identified factors associated with APD to be the instance when a patient requires substantial support for the activities of daily living, motor fluctuations or dyskinesia, falls, freezing of gait, as well as the presence of the non-motor symptoms orthostatic hypotension, excessive daytime somnolence, dementia, or neuropsychiatric deficits.

Not all patients with PD progress to APD, highlighting the urgent need to identify those at risk of developing this debilitating stage (Martínez-Castrillo et al. 2021). The pathophysiology of APD remains incompletely understood, posing challenges for early detection and intervention. As the APD stage requires intensified treatment and limits the quality of life and prognosis, there is a pressing demand for biomarker-supported approaches to predict critical milestones in disease progression. These include the onset of motor fluctuations, cognitive decline, and the variability in therapeutic response to advanced interventions such as continuous infusion therapies or deep brain stimulation. These clinical manifestations of APD are closely linked to regional patterns of neurodegeneration, emphasizing the importance of understanding the underlying pathophysiology (Schröter et al. 2022). Neuroimaging holds great promise in this context, offering the potential to not only elucidate the mechanisms driving APD but also identify risk factors or compensated stages earlier in the disease course.

Concurrently, modern imaging techniques are being used to define structural and functional changes associated with each of the individual motor and nonmotor symptoms traditionally associated with APD (Ryman and Poston 2020). While nuclear medicine techniques such as single-photon emission computerized tomography (SPECT) and PET positron emission tomography (PET) have been instrumental in identifying both indirect and direct molecular changes associated with PD progression(McCluskey et al. 2020; Mitchell et al. 2021; Bohnen et al. 2022), conventional magnetic resonance imaging (MRI) provides valuable insights into macrostructural abnormalities such as atrophy in key brain regions, indicative for copathologies and broadly applied in clinical routine in the differential diagnosis of PD (Ryman and Poston 2020). More advanced MRI techniques have the potential to capture microstructural changes that are more closely linked to the pathophysiology of PD (Qi et al. 2025). Table 1 provides an overview including advantages and disadvantages of the most frequently applied MRI techniques to study PD-related alterations of the brain.

**Table 1:** Overview of the most frequently applied MRI techniques to study PD-related alterations of the brain

MRI Method	Advantages	Disadvantages
**T1-weighted Imaging**	High-resolution anatomical and morphometric information allows for detecting structural atrophy. Frequently employed as an anatomical reference for other neuroimaging modalities.	Limited sensitivity to early neurodegenerative alterations such as microstructural changes. Some neurodegenerative diseases do not exhibit a pathognomonic pattern of atrophy in T1w.
**T2-weighted Imaging**	High-contrast information on e.g. gliotic tissue alterations such as microvascular lesions	Limited specificity for distinguishing origin of signal alteration, as T2w hyperintensities can result from various non-specific pathologies (e.g., small vessel disease, aging, demyelination).
**Susceptibility-Weighted Imaging (SWI)**	Highlights neurodegeneration-induced iron accumulation or atrophy of physiologically iron-containing structures such as the substantia nigra.	Limited specificity for the diagnosis of neurodegenerative Parkinson syndromes and cannot differentiate PD from other neurodegenerative disorders regarding nigral degeneration.
**Quantitative Susceptibility Mapping (QSM)**	Provides quantifiable imaging of iron content changes.	Susceptible to artifacts from air-tissue interfaces and calcifications; interpretation can be affected by orientation dependency and background field effects.
**Neuromelanin Imaging**	Detects pigment loss in the substantia nigra, as a quantitative biomarker for neurodegeneration.	Limited availability and requires specialized acquisitions.
**Diffusion Tensor Imaging (DTI)**	Assesses microstructural integrity based on the diffusion properties of the brain tissue. Facilitates tractographic approaches that allow investigation of changes in the structural connectome.	Especially developed for white matter analyses. Alterations in DTI-derived parameters can be challenging to interpret.
**Diffusion-Based Multicompartment Imaging**	More advanced diffusion-weighted acquisitions such as multishell imaging can provide biophysically motivated information on the microstructural cellular integrity and intracellular water distribution across different mesostructural compartments. This technique is better applicable than DTI to grey matter structures and provides more interpretable parameters.	Requires more advanced postprocessing and some approaches such as Neurite Orientation Dispersion and Density Imaging cannot reliably detect severe microstructural alterations as they are hampered by hard a priori constraints in parameter modelling.
**Functional MRI (fMRI)**	Investigates blood desoxygenation to evaluate brain activity. This allows to reveal changes in functional connectivity both in the resting state but also task-specific.	Interpretation can be complex due to the unknown exact underlying source of the signal; influenced by patient movement.

Current efforts are ongoing for the development of PET techniques allowing for the direct imaging of pathological protein aggregates such as alpha-synuclein, tau, and beta-amyloid linked to PD and important copathologies such as Alzheimer`s disease. This narrows the gap between the underlying pathological changes and in vivo presentation, offering insights into the underlying molecular mechanisms or potential target engagement in the evaluation of new disease-modifying therapies (Petrou et al. 2015; Zhang et al. 2023; Endo et al. 2024). Furthermore, PET can detect disease-typical metabolic alterations using tracers such as 18 F-fluorodeoxyglucose (FDG). These alterations in cerebral metabolism often precede the onset of clinical symptoms by years, providing an early window into disease progression and facilitating disease staging (Meyer et al. 2017). While conventional MRI performs well in many neurodegenerative diseases, including Alzheimer’s or frontotemporal dementia, it does not identify neurodegeneration in PD with sufficient sensitivity and specificity (Heim et al. 2017). Recent developments in advanced MRI techniques allowed for quantifying pathological alterations in the cerebral tissue in PD. For instance, neuromelanin-specific imaging facilitates the assessment of the iron content in the substantia nigra (Samson and Noseworthy 2022) and diffusion-based techniques allow for the investigation of the cerebral microstructural integrity.

### Neuroimaging in the differential diagnosis of advanced Parkinson’s disease

In APD, the condition has been present for a long time. This makes the differential diagnosis of PD frequently less important than at the earlier disease stages. However, there is a growing presence of comorbidities, and imaging has ample potential in assessing them. The development of novel and further refinement of established imaging techniques is transforming the differential diagnostic approach to APD. As the deposition of alphaSynuclein aggregates is one of the earliest manifestations of PD, alpha-Synuclein-PET tracers are promising for disease staging and might identify risk constellations of binding patterns. Recently, different tracers were investigated and allowed for delineating Parkinson syndromes based on their underlying pathology. However, only 18 F-C05-05 has a noteworthy binding in PD, and the value of these tracers needs to be addressed in clinical studies (Smith et al. 2023; Endo et al. 2024). Notably, no current suitable PET ligands targeting alpha-synuclein in the human brain are clinically established (Xiang et al. 2025). Tau-PET allows for the exclusion of tauopathies in the differential diagnosis of neurodegenerative Parkinson syndromes as well as for the assessment of tau copathology in PD-associated dementia (Brendel et al. 2020; Tang et al. 2023). Furthermore, Alzheimer`s disease copathology can be assessed via amyloid-PET and might facilitate predicting the rate of cognitive decline (Gomperts et al. 2013; Petrou et al. 2015).

Conversely, FDG-PET does not indicate the presence of pathological proteins but reveals pathological cerebral metabolism. It is established in the differential diagnosis of Parkinson syndromes, with a sensitivity and specificity of around 90% as different entities show distinct patterns of hypometabolism (Meyer et al. 2017). In the context of PD, its particular diagnostic strength is the detection of parieto-occipital hypometabolism. This indicates cortical involvement and can be utilized either in the differential diagnosis of PD or disease staging. Beyond this, metabolic network analysis might improve the differential diagnosis of neurodegenerative Parkinson syndromes (Eckert et al. 2005; Schindlbeck and Eidelberg 2018). In addition, PET tracers for specific neurotransmitter systems, such as 11 C-PMP for acetylcholinesterase activity, can also be used in the differential diagnosis of PD and have shown promising results; however, their translation into clinical routine has not yet occurred. (Gilman et al. 2010).

MRI is well-established and widely used in clinical practice to rule out alternative causes of Parkinson syndromes such atypical Parkinson syndromes or normal pressure hydrocephalus, and white matter lesions, though the latter two can be potential copathologies, thereby aiding in establishing the diagnosis of PD (Saeed et al. 2020; Schröter et al. 2023). Additionally, macroatrophy in MRI can predict the presence of Alzheimer’s disease copathology (ten Kate et al. 2018). However, the diagnostic value in early-stage PD is limited as no pathognomonic signal alteration is present, and macrostructural atrophy typically manifests in the late stages of PD (Menke et al. 2014), however, without a specific pattern. More advanced MRI techniques facilitate the detection of alterations in the cerebral integrity beyond macrostructural atrophy. In particular, neuromelanin-sensitive imaging allows for assessing the iron content of brain regions such as the substantia nigra and has demonstrated a sensitivity and specificity of around 80% in identifying PD according to a recent meta-analysis (Wang et al. 2019).

In addition, diffusion-weighted MRI has shown substantial diagnostic potential in the detection of PD, as it provides information on the cerebral microstructure. A recent international multicentre study using machine learning techniques highlighted the high diagnostic performance of novel diffusion-weighted MRI approaches with areas under the receiver operating curves larger than 0.9 (Archer et al. 2019; Schröter et al. 2024a). Furthermore, modern multi-shell diffusion techniques that rely on multi-compartment models such as Neurite Orientation Dispersion and Density Imaging (NODDI) (Mitchell et al. 2022) or Diffusion Microstructure Imaging (DMI) offer promising tools for detecting subtle disease-related changes such as increased nigral or putaminal free interstitial fluid in PD as a surrogate for neurodegeneration (Schröter et al. 2022). Despite the potential of neuroimaging to capture neurodegenerative alterations in vivo and quantify them, the heterogeneous and complex symptom patterns of APD do not result from a comprehensive pattern of neurodegeneration. Thus, no APD pattern in neuroimaging has been established so far. This might be attributed to a variable affection of distinct systems that each individually contribute to another symptom complex in APD. Here, neuroreceptor-specific molecular imaging might facilitate an insight into the pathophysiology. Given the proven iteration of imaging biomarkers from different modalities, each highlighting different aspects of the pathology, a promising solution for the differential diagnosis of PD and Parkinsonian syndromes is the synergistic use of PET/MRI acquisitions. Hybrid PET/MRI systems allow the concurrent acquisition of molecular, structural, and functional data, posing direct positive effects on efficiency while minimizing the inaccurate spatial alignments across modalities and facilitating image segmentation. In this regard, elevated levels of glucose consumption (FDG) uptake in the striatum of early PD patients has been reported when compared to MSA, while MSA displays hypometabolism in the putamen, pons, and cerebellum (Hu et al. 2021). Conversely, given that PD and LBD are known to present similar patterns on FDG PET/MRI, a combination of different tracers may be necessary for different parkinsonian syndromes. For example, dopaminergic synthesis and transmission tracers, e.g., FDOPA-PET or the presynaptic transporter-targeting (FP-CIT-PET)– see Leung and Strudwick for a more detailed review on this topic (Leung and Strudwick 2024).

While APD defines a stage with heterogeneous clinical presentation, the insight into the pathophysiological process underlying individual hallmark symptoms might allow for a better understanding, identifying patients at risk and potentially revealing treatment targets as well as offering great potential in the biomarker-based staging of PD.

### Neuroimaging associated with motor fluctuations in advanced Parkinson’s disease

Motor fluctuations represent a debilitating complication in APD. A comprehensive understanding of the underlying mechanisms of these fluctuations is imperative for enhancing patient management and developing targeted therapies. Imaging techniques capable of capturing subtle neurodegenerative changes hold considerable potential for the identification of biomarkers that can facilitate the diagnosis, staging, and characterization of motor fluctuations. Neuromelanin-sensitive MRI has shown that neuromelanin content in the substantia nigra is significantly reduced in patients with motor fluctuations compared to those without. This finding suggests a possible link between neuromelanin loss and the development of these motor complications (Okuzumi et al. 2019). Interestingly, this difference was not associated with significant variation in dopamine transporter (DAT) availability, suggesting that neuromelanin-sensitive imaging may capture aspects of nigral degeneration not reflected by DAT imaging. A second study further validated this observation by comparing patients undergoing evaluation for deep brain stimulation for motor fluctuations with de novo PD patients. The reduction in neuromelanin content was particularly pronounced on the side contralateral to the clinically more affected side of the body (Vitali et al. 2020). In summary, neuromelanin-sensitive MRI has the potential to serve as a biomarker for motor fluctuations in APD, offering insights into aspects of nigral degeneration that extend beyond the scope of dopamine transporter imaging. More insight into the pathophysiolocy and neurochemistry is feasible via neuroreceptor-specific PET. While Fuente-Fernández attribute motor fluctuations to an altered levels of synaptic dopamine using 11 C-raclopride PET (de la Fuente-Fernández et al. 2001), Laurencin and colleagues implicated noradrenergic demise to be associated with motor fluctuations (Laurencin et al. 2024). Further research should investigate the affection of underlying networks or metabolism patterns.

### Neuroimaging associated with dyskinesia in advanced Parkinson’s disease

Levodopa-induced dyskinesia (LID) significantly impacts patients’ quality of life and profoundly influences therapeutic strategies. The putaminal dopamine turnover, as quantified by 18 F-dopa PET, has shown promising potential for the prediction of future dyskinesia, as an elevated putaminal dopamine turnover in de novo PD was associated with an increased risk of developing later motor complications (Löhle et al. 2016). Several PET studies have investigated the role of serotonergic mechanisms in LID by assessing striatal dopamine release. Here, a relative preservation of serotonin transporter binding in the putamen was observed in PD patients with LIDs. Additionally, identical levodopa doses resulted in significantly higher striatal synaptic dopamine concentrations in patients with LIDs compared to those with stable responses to levodopa, highlighting the role of serotonin in dyskinesia in PD (Politis et al. 2014; Pagano et al. 2017). Furthermore, opioid receptor PET revealed reduced striatal and thalamic opioid receptor density in patients with dyskinesia, indicating dyskinesia in PD to originate from degeneration in multiple neurotransmitter systems (Piccini et al. 1997).

A recent MRI study comparing PD with and without LID observed altered perivascular spaces, globally as well as regionally in the right inferior frontal gyrus, also showing a strong association with symptom severity (Cao et al. 2024). Furthermore, Su and colleagues have revealed that patients with LID exhibit reduced neuromelanin in the substantia nigra pars compacta compared to those without LID. Conversely, quantitative susceptibility mapping revealed increased iron deposition in the same localization attributable to regional neurodegeneration. Interestingly, patients with LID also have a higher microstructural intracellular volume fraction, which is thought to reflect compensatory axonal changes consistent with findings from neuropathological studies (Su et al. 2023). Differences in white matter isotropic diffusion and neurite density were identified between PD with and without LID, particularly in tracts like the external capsule and inferior longitudinal fasciculus, with more pronounced degeneration in patients without LID. These findings suggest that dysbalanced degeneration in temporal fibers may contribute to the pathogenesis of LID in PD (Ogawa et al. 2021). In sum, multimodal imaging revealed alterations in dopamine turnover, serotonergic dysfunction, and structural changes in substantia nigra and white matter tracts to be associated with LID. These findings emphasize the complex neurobiological mechanisms contributing to the pathophysiology of LID and underscore the potential of modern imaging techniques for predicting and understanding this debilitating complication.

### Neuroimaging associated with therapy response to deep brain stimulation in advanced Parkinson’s disease

When patients develop motor fluctuations or LID, continuous therapeutic approaches are often implemented, with pump-based therapies and deep brain stimulation being commonly used. These treatments aim to provide more stable symptom control and reduction of LID. In contrast to other device-based therapies, such as pump-based devices, the response to DBS can only be indirectly assessed by the response to levodopa before surgery (Schröter et al. 2024b). A reliable, non-invasive biomarker for predicting response to deep brain stimulation is therefore urgently needed. FDG-PET showed promising results as a predictor of postoperative apathy after deep brain stimulation of the subthalamic nucleus (Gesquière-Dando et al. 2015). The extent of white matter hyperintensities is inversely associated with the response to deep brain stimulation (Cavallieri et al. 2021). In addition, volumetric MRI studies using a voxel-based approach have produced heterogeneous results, suggesting that grey matter atrophy in frontoparietal regions as well as the right thalamus correlate with motor response to deep brain stimulation (Yim et al. 2020; Jergas et al. 2023). However, no association was found between grey matter atrophy and improvement in non-motor symptoms (Loehrer et al. 2024b). In terms of cerebral microstructure, microstructural integrity of the subthalamic nucleus and substantia nigra was recently reported to be associated with motor improvement (Hermann et al. 2024). Alleviation of non-motor symptoms was predicted by microstructural integrity of the insular cortex, putamen, or cingulum in another diffusion-weighted MRI study (Loehrer et al. 2024a). In summary, several studies have identified potential biomarkers for predicting motor and non-motor responses to deep brain stimulation in PD. However, effect sizes were not sufficient for single-patient level prediction of outcome and thus, neuroimaging findings might rather be integrated into multifaceted scoring systems, but these findings require further validation for clinical application.

### Advanced imaging associated with dementia in advanced Parkinson’s disease

Cognitive deficits are frequent in PD, with up to 80% developing dementia within 20 years after onset (Hely et al. 2008). In cognitive impairment, Alzheimer’s disease copathology is frequently observed and associated with a rapid cognitive decline (Aarsland et al. 2021). Advanced imaging allows for identifying neurodegeneration and preceding protein depositions in regions associated with cognition in patients with and without impaired cognition. These techniques have greatly improved our understanding of cognitive deficits in PD, particularly in the transition from mild cognitive impairment (PD-MCI) to Parkinson’s disease dementia (PDD). Tau- and amyloid-PET imaging have revealed increased cortical tau and cortical as well as subcortical beta-amyloid binding in PDD compared with PD-MCI and cognitively unimpaired PD patients (Petrou et al. 2015; Shah et al. 2016; Tang et al. 2023).

Another promising approach is to measure neuroinflammation, using tracers like TSPO-PET for microglia, 11 C-BU99008 PET for astrocytes, or P2 × 7R for the inflammasome, among others (Jain et al. 2020; Kreisl et al. 2020).

Patients at higher risk of developing PDD show increased inflammation in regions such as the hippocampus, amygdala, substantia nigra and putamen compared to those at lower risk (Kouli et al. 2024). Furthermore, studies utilizing FDG-PET, such as those by Gonzáles-Redondo et al., have shown that parieto-occipital cortical hypometabolism precedes grey matter atrophy observed on MRI, highlighting its potential role in the early detection of patients at risk of cognitive decline (González-Redondo et al. 2014). Utilizing 11 C-UCB-J, a tracer with affinity to the presynaptic vesicle protein SV2A to assess synaptic density revealed a widespread cortical reduction in synaptic density in PDD and DLB but not in PD without cognitive impairment. As tracer uptake was associated with cognitive impairment, this might potentially serve as a biomarker for disease severity (Andersen et al. 2021; Martin et al. 2024). Complementary, previous molecular imaging studies have reported widespread cortical cholinergic deficits in patients with PDD, associated with cognitive impairment (Bohnen et al. 2003; Klein et al. 2010).

MRI has a major strength in the evaluation of pathologies known to be associated with impaired cognition, such as severe microangiopathy or normal pressure hydrocephalus (Heim et al. 2017; Rau et al. 2021). This is of relevance as these conditions can be therapeutically addressed. In addition, microstructural changes in the basal forebrain have been associated with cognitive decline in PD (Schulz et al. 2018). MRI has successfully been employed for the prediction of PD-related cognitive impairment, with studies showing that reduced corpus callosum volume (Goldman et al. 2017) and reduced cortical thickness in the caudal anterior cingulate cortex and parieto-occipitotemporal cortices are associated with a higher risk of cognitive decline (Filippi et al. 2020; Gorges et al. 2020). Moreover, advanced microstructural MRI allows for disentangling the differential contribution of regional neurodegeneration to disctinct cognitive subdomains (Rau et al. 2025). In summary, modern imaging techniques, encompassing tau-, amyloid- and TSPO-PET to assess Alzheimer’s disease copathology and neuroinflammation, along with FDG-PET have demonstrated efficacy in the identification of early neurodegenerative alterations associated with cognitive decline in Parkinson’s disease. In addition to its traditional strength in detecting comorbid pathologies such as white matter lesions, advances in new sequences and analysis techniques are expanding the potential of MRI for investigating the pathophysiology and clinical staging of cognitive deficits. These biomarkers are associated with diminished cognitive performance and may facilitate the prediction of the transition from PD-MCI to PDD; however, further validation is necessary to substantiate these findings.

### Imaging findings associated with orthostatic hypotension in Parkinson’s disease

Orthostatic hypotension is a common occurrence in APD, which strongly contributes to reduced mobility, lower social participation, and the risk of falls. Orthostatic hypotension in PD is attributed to postganglionic sympathetic damage. The myocardial denervation frequently observed in PD-related orthostatic hypotension is best assessable with nuclear medicine imaging such as 123I-metaiodoben-zylguanididine scintigraphy (Jost et al. 2010) or 18 F-Fluorodopamine PET (Goldstein et al. 2000). PET can moreover detect extracardiac denervation, e.g. of the renal cortex and the thyroid gland that can contribute to impaired orthostasis, too (Tipre and Goldstein 2005). Other traces, particularly 11 C-HED-PET, have been used to assess cardiac sympathetic denervation in PD, indicating significant heterogeneity of cardiac sympathetic neural integrity (Wong et al. 2017).

### Neuroimaging associated with excessive daytime somnolence in Parkinson’s disease

Several transmitter systems have been employed to study excessive daytime somnolence (EDS) in PD. The extent of the dopaminergic deficits is associated with the presence of EDS as well as with EDS severity (Yousaf et al. 2018). Furthermore, thalamic presynaptic monoamine transporter density is reduced in patients with EDS, showing an inverse relationship between symptom severity and monoamine transporter density, implicating not only the dopaminergic but also the serotonergic system to be involved in EDS (Yoo et al. 2020).

Several MRI studies investigated EDS in PD using various techniques. Volumetric analysis revealed increased volumes in mesiotemporal regions. This was accompanied by altered white matter integrity in various tracts (Chondrogiorgi et al. 2016). In contrast, Kato and colleagues found widespread gray matter atrophy in EDS with accentuation of the basal forebrain, indicating a cholinergic degeneration (Kato et al. 2012). Surface analyses noted both increased cortical and contracted subcortical surfaces in EDS (Rosinvil et al. 2024). The affection of limbic pathways, however, was observed by tractographic studies in PD-related EDS, too (Wen et al. 2016; Ashraf-Ganjouei et al. 2019).

Functional MRI revealed the impairment of wake-promoting pathways and the default mode network in excessive daytime sleepiness (Wang et al. 2020) as well as both up- and downregulation in different networks (Wen et al. 2016) or distinct brain regions such as the left angular gyrus (Zheng et al. 2023). Though neuroimaging provided insight into the pathophysiology of excessive daytime somnolence in PD, most studies were conducted with rather small single-center collectives, and the prognostic value of cerebral alterations has not yet been investigated.

### Neuroimaging associated with survival in advanced Parkinson’s disease

Dementia in PD is strongly associated with worse prognosis and reduced survival. Recent studies have highlighted the predictive value of advanced imaging in this context. For example, Brumberg et al. (2024) corroborated a previous study based on a small sample size that indicated that the severity of parieto-occipital hypometabolism, a hallmark of cognitive deficits, correlated with survival (Hellwig et al. 2015; Brumberg et al. 2024). Patients with moderate cortical involvement had a median survival of less than seven years, whereas patients with severe cortical involvement had a median survival of less than five years. These findings highlight the role of advanced imaging biomarkers not only in characterizing disease progression but also in informing prognosis and possibly guiding clinical decision-making, e.g., when deep brain stimulation is considered.

### Future directions in neuroimaging in advanced Parkinson’s disease

As research criteria for APD were just recently published, a precise investigation of the APD stage beyond individual symptoms is yet lacking in imaging studies. APD presents a highly heterogeneous clinical spectrum with distinct phenotypes that remain poorly characterized and understood. Future neuroimaging research should aim at refining the classification of these phenotypes, allowing for a deeper understanding of the underlying pathophysiology, and providing objective biomarkers for subtyping, clinical decision-making as well as prognosis. A comprehensive understanding of the mechanisms driving specific symptom constellations will not only improve clinical decision-making but also facilitate the development of targeted therapies. Neuroimaging is best suited for this, as it allows for the investigation of the symptom trajectories in vivo. Additionally, neuroimaging allows for the identification of patients at risk of progression to APD before clinical manifestations occur. Such prodromal identification would allow for timely intervention, improved patient stratification, and personalized therapeutic strategies, improving quality of life and reducing the risk for complications.

As aforementioned, a promising path for evaluating pathophysiological mechanisms of PD is the use of the synergistic PET/MRI data. For example, PET/MRI studies have revealed an early and uneven dopaminergic impairment within the putamen, accompanied by disruptions in the integrity of the nigrostriatal pathway in PD compared to healthy individuals. Moreover, the reported microstructural alterations were closely linked to diminished motor performance, driven by molecular degeneration in the putamen (Shang et al. 2021). Thus, the combination of different modalities may offer mechanistic insight into pathological and clinical features.

## Conclusion

In the midst of a Parkinson’s pandemic (Dorsey et al. 2018), the relevance of APD is drastically increasing. However, many challenges have yet to be solved, such as the treatment of dyskinesia, the treatment of cognitive deficits, ideal patient selection for continuous therapies, or prognostication of survival. While neuroimaging already offers potential solutions for these challenges in APD, most of these approaches lack validation in well-defined, prospective cohorts or have only been applied in the earlier disease stages, thus hampering the translation of these findings to APD. It is imperative to address these questions by conducting rigorous, well-designed, prospective studies and validating neuroimaging approaches in APD to bridge the gap between research and clinical application. Additionally, further work combining MRI and PET modalities, including data coming from hybrid PET/MRI techniques, may shed light onto changes at different scales of the pathology.
